# Genetic Algorithm Approach to the 3D Node Localization in TDOA Systems

**DOI:** 10.3390/s19183880

**Published:** 2019-09-09

**Authors:** Javier Díez-González, Rubén Álvarez, David González-Bárcena, Lidia Sánchez-González, Manuel Castejón-Limas, Hilde Perez

**Affiliations:** 1 Department of Mechanical, Computer, and Aerospace Engineering, Universidad de León, 24071 León, Spain (L.S.-G.) (M.C.-L.) (H.P.); 2 Positioning Department, Drotium, Universidad de León, 24071 León, Spain; 3 IDR/UPM Universidad Politécnica de Madrid, Pozuelo de Alarcón, 28223 Madrid, Spain

**Keywords:** genetic algorithm, LPS, Asynchronous, TDOA, CRLB, sensor networks, passive localization

## Abstract

Positioning asynchronous architectures based on time measurements are reaching growing importance in Local Positioning Systems (LPS). These architectures have special relevance in precision applications and indoor/outdoor navigation of automatic vehicles such as Automatic Ground Vehicles (AGVs) and Unmanned Aerial Vehicles (UAVs). The positioning error of these systems is conditioned by the algorithms used in the position calculation, the quality of the time measurements, and the sensor deployment of the signal receivers. Once the algorithms have been defined and the method to compute the time measurements has been selected, the only design criteria of the LPS is the distribution of the sensors in the three-dimensional space. This problem has proved to be NP-hard, and therefore a heuristic solution to the problem is recommended. In this paper, a genetic algorithm with the flexibility to be adapted to different scenarios and ground modelings is proposed. This algorithm is used to determine the best node localization in order to reduce the Cramér-Rao Lower Bound (CRLB) with a heteroscedastic noise consideration in each sensor of an Asynchronous Time Difference of Arrival (A-TDOA) architecture. The methodology proposed allows for the optimization of the 3D sensor deployment of a passive A-TDOA architecture, including ground modeling flexibility and heteroscedastic noise consideration with sequential iterations, and reducing the spatial discretization to achieve better results. Results show that optimization with 15% of elitism and a Tournament 3 selection strategy offers the best maximization for the algorithm.

## 1. Introduction

Global Positioning Systems (GNSS) have been traditionally used to guide vehicle navigation in outdoor environments. However, the accuracy achieved by these systems has been insufficient for some tasks such as the navigation of AGVs, UAVs, precision agriculture, surveillance and espionage work, or indoor location of vehicles. For this reason, over the last few years, local positioning systems (LPS) have been developed where the proximity between their sensors and the positioning targets allows for the significant reduction of errors in positioning.

Both the GNSS and the LPS make measurements of certain parameters of the signals such as time [1], frequency [2], or power [3] in order to determine the location of the vehicles. From these models, the most popular are those based on temporary measurements because of their simplicity, robustness, accuracy, and ease of implementation. Among these temporary systems, it is possible to find the Time of Arrival (TOA) [4] and Time Difference of Arrival (TDOA) systems [5].

The TOA systems are based on the measurement of the absolute times of travel of the signal between the transmitter and the receiver. This requires synchronization between all the sensors involved in the calculation of the position, including the target. On the other hand, the TDOA systems are based on the measurement of the relative flight times of the signal reaching two different receivers.

In this case, the synchronization of the sensors is optional since the computation of the time differences can be computed in a single clock in a coordinating sensor [6], as in the architecture A-TDOA [7]. The elimination of the synchronism in A-TDOA produces a global reduction of the positioning error and an increase in the stability of the position calculation over the time due to the elimination of the initial time offset and the drift [8], as these factors introduce errors in this methodology. Even though both TOA and TDOA systems can achieve better peaks of accuracy, the overall accuracy of these systems is reduced since the last synchronization of their clocks, showing the A-TDOA architecture better results and stability over the time. As a consequence, these asynchronous architectures are taking on a special relevance at present in applications that require high accuracy in positioning.

There is also a distinction in the systems according to the function of the target in the processing of the positioning signal. The systems in which the position is calculated in a receptor of the vehicle, with the system nodes acting as emitters, are considered as direct or centralized methods, while the systems where the vehicle only sends the signal to the receiver nodes are considered as passive or decentralized methods. In this paper, the passive A-TDOA architecture has been selected according to its accuracy in highly demanded applications.

However, once the positioning architecture has been selected, along with the algorithms that allow for the calculation of the position [9,10,11], the only factor that allows the reduction of the global positioning error of the vehicles is the spatial distribution occupied by the sensors or satellites in space with respect to positioning targets. This spatial distribution strongly affects the system accuracy by increasing the positioning errors due to the changes in the geometric properties of the intersection of the surfaces containing the possible locations of the targets in the space [11] -spheres in TOA systems and hyperboloids in TDOA systems. An inappropriate sensor deployment can also produce an increase in the quantity of the noise received in the nodes and an increase of the multipath generated during the transmission. All these factors can be controlled through an optimized node localization.

This problem has been widely studied in recent years, although it differs significantly depending on the location of satellites in Global Navigation Satellite Systems (GNSS) such as GPS, GLONASS, or Galileo and local positioning networks.

GNSS positioning systems seek global coverage as the main design requirement. This has led to the location of constellations of satellites that are at the same height above the earth’s surface. As a consequence, the signals emitted by the satellites travel practically the same distance until they reach the positioning targets and their signals suffer distortions that could be considered homogeneous. In addition, the high cost of its satellites has promoted the search for the minimization of the deployment of satellites in space to reduce the overall costs of these systems. The satellites are all dependent on each other since the requirements of achieving a global synchronism of the positioning system significantly reduce the flexibility of the design.

On the other hand, LPS seek to maximize the accuracy in determining the position and thus they use an adequate number of sensors to reduce the errors in order to meet their needs. The altitudes in which the sensors are located are variable and highly dependent on the characteristics of the ground surface where they will be placed. The variability of the distances between the target and the different sensors causes the heteroscedasticity of the noise measured in the sensors [12]. In addition, in LPS networks, sensors act in a more independent way since not all of them have to be interconnected with each other, and even asynchronous architectures are being developed [7,8] which increase the flexibility of these systems. As a consequence, it is necessary to establish a methodology to optimize the location of the beacons in LPS systems, considering the special characteristics of these architectures.

## 2. State of Art

The first studies on sensor location in Local Positioning Systems were focused on the minimization of the number of sensors displayed. Francis et al. [13] demonstrated that the initial linear models used to solve the node distribution problem were excessively complex. This hypothesis was supported in the large dimensionality of the solutions space, and it is the reason why he proposed a reduction of the complexity based on a grid model. Shoval et al. [14] modified the conception of the problem with the consideration of the C-N-LNR (Continuous non-linear) model in order to compute an optimization through a greedy-type algorithm. In this model, sensor location is defined between two main areas: maximum area (effective coverage of the sensor) and minimum area (where the least possible separation between sensors is considered). C-N-LNR aims for an optimization in the node distribution with special consideration of some critical points in the AGVs navigation.

The usage of heuristic methods has proven to be especially suitable for the sensor location problem. Tekdas et al. [15] and subsequently Yoon et al. [16] concluded that this distribution problem is NP-hard, which led to a heuristic method solution such as Genetic Algorithms (GA) [11,17,18,19,20,21,22,23,24], simulated annealing [25] or Tabu search methodologies [26].

The initial solutions of the problem through GA were based on Global Dilution of Precision (GDOP) as the factor to measure the quality of the sensor locations [27,28]. GDOP has been widely used in Global Positioning systems. GDOP consists of a measurement of the suitability of the relative position between the target and the sensor location, Position Dilution of Precision (PDOP), and the uncertainty of time measurements, Time Dilution of Precision (TDOP). While the TDOP depends on the measuring instruments, PDOP is a geometric factor which is determined by the calculation of the volume defined by the unitary vectors of the target with each sensor. The minimum value of the PDOP strongly depends on the number of sensors of the distribution [29].

Nevertheless, GDOP is constructed under the assumption of similar distances between target and nodes, which happens only in satellite navigation. However, LPS can have variable distances in the target-node connection, leading to different noises in the signal receivers. Burke et al. [30] justified that noise variances can vary notably among sensors so that a heteroscedastic model in the time measurements must be considered to achieve practical results [31].

This model is evaluated in this paper through the CRLB, which is an unbiased estimator of the lowest uncertainty in a positioning system under Line-of-Sight (LOS) and Non-Line-of-Sight (NLOS) conditions [32].

A path loss propagation model is introduced in order to consider signal depreciation for an asynchronous positioning architecture (A-TDOA) for the first time. Furthermore, a 3D positioning of the automatic vehicles, both AGVs and UAVs, is considered in an LPS. This factor presents a novelty in comparison with the other papers considered in this revision, which are focused on 2D positioning. This consideration forces the introduction of a third coordinate as a new degree of freedom in the sensor location in the space.

In conclusion, this paper presents a 3D optimization in the sensor location of an asynchronous positioning architecture through the CRLB with heteroscedastic noises for the first time in the literature according to our best knowledge. An actual 3D environment has been defined to locate the sensors and to allow for navigation of the vehicles. The optimization process is performed via a genetic algorithm that will be presented in the paper.

The remainder of the article is organized as follows: In Section 3, an actual ground model is presented and the main area for navigation of the vehicles and location of the sensors are defined; in Section 4 the genetic algorithm characteristics are presented and its usage is justified. The evaluation of the quality of the node distributions in the genetic algorithm by means of CRLB is analyzed in Section 5. Section 6 shows the results that are then discussed in Section 7. And finally, Section 8 presents the conclusions related to the research reported in this paper.

## 3. Ground Model

The proposed GA allows the 3D optimization of the positioning sensor placement based on any base surface characterization, regardless of fluctuations in the elevation of the modeling environment. Additionally, this GA admits the generation of any optimization region at the base surface level (AGV modeling) or above it (UAV modeling).

**Figure 1:**
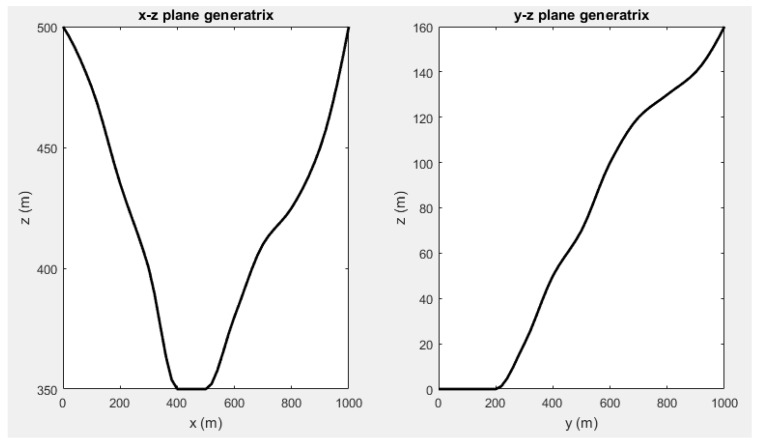
Base surface elevation profiles expressed in terms of *x*-*z* and *y*-*z* plane generatrix.

The flexibility of the presented methodology is proven via the characterization of an irregular 1000 × 1000 m base surface for all analysis optimizations. This modeling is defined by the projections of the ground curves on the *x*-*z* and *y*-*z* planes, which are shown in Figure 1. In addition, base surface elevation has been distorted with a normal distribution N(5, 1) m.

The characterization of the scenario is completely defined with two regions: Node Localization Environment (NLE) and Target Localization Environment (TLE). NLE is the space where nodes have free possible movement during genetic algorithm performance. The projection of the NLE to the base surface can have an area equal to or smaller than this, and there may be obstacle zones where the nodes cannot be located. Regarding elevation of the NLE, it is defined based on maximum and minimum elevations with respect to the modeling surface. The minimum pre-set elevation is 3 m with the aim of avoiding adverse phenomena originated by multipath trajectories that degrade the final position estimations. The maximum elevation of the nodes has been established as 10 m above the base surface, in order to limit the impact of the use of large supports to place the sensors.

TLE defines the entire space of possible locations of the targets that are going to be positioned. It is possible to perform its modeling both in the projection on the base surface and in elevation, which will be dependent on the type of optimization carried out. The lower limitation in height depends on the application to be optimized, while the upper limit will be as a general rule of 120 m (maximum flight height for UAVs).

Next, we present the two scenarios in which the final optimization of the sensor distribution will be based on the CRLB. The spatial discretization of the TLE region is 30 m in Cartesian coordinates x-y and 5 m in the *z*-coordinate, in order to achieve the compromise solution between spatial resolution and processing time, taking advantage of the continuity of the accuracy results obtained when fine grids are implemented. In the case of the NLE, due to the adaptability of the length of chromosomes based on the region limits and the GA properties, the spatial resolution varies in the three coordinates from 0.5 to 1 m. In addition, if lower spatial resolutions are required, it could be achieved by the GA through the modification of the length of chromosomes to a higher value than initially determined.

**Figure 2:**
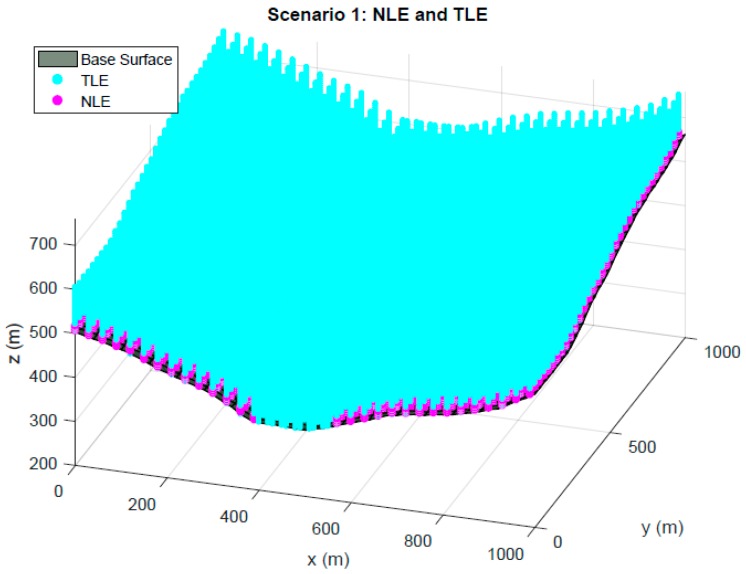
Scenario 1. First environment characterization for optimization with Genetic Algorithms (GA).

Figure 2 shows the first of the scenarios submitted to the optimization process. The plan projection of the NLE and the TLE presents an area equal to the base surface, with the elevation limitations previously defined.

**Figure 3:**
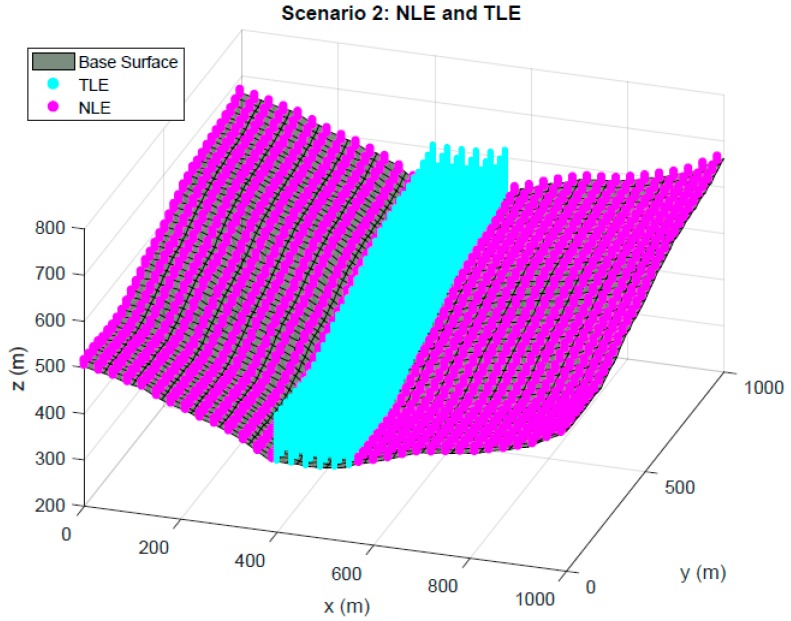
Scenario 2. Second environment representation for optimization with Genetic Algorithms –(GA). TLE region is limited to the center of the domain. NLE space extends all over the base surface, except for TLE region.

Figure 3 shows the second of the scenarios under analysis. In this case, the optimization is carried out for a TLE that extends to a bounded region of the base surface, fulfilling the limitations in elevation for Scenario 1. The NLE occupies the same volume as in Scenario 1, except for the space occupied by the TLE, which on this occasion cannot be used for the location of nodes inside it.

## 4. Genetic Algorithm

The total space of solutions of the optimization problem is dependent on the degree of spatial resolution required in the location of the nodes, which in turn will be determined by the spatial discretization developed on the NLE regions. For positioning of **n** sensors and with the spacings specified above, there will be 4624*^n^* and 3944*^n^* possible solutions to the problem in Scenario 1 and 2 respectively.

The magnitude of this search space prevents the use of exact resolution techniques that examine the entire spectrum of solutions to achieve the optimization of the problem in question. Additionally, greedy resolution techniques and those based on a recursive division [33] of the problem are discouraged due to the great joint dependence between the location of the different nodes and the final solution.

These aspects lead to the optimization of this problem being carried out through heuristic techniques [34]. Among them, genetic algorithms present multiple advantages, among which are great flexibility and robustness, use of non-derivable fitness functions, parallel treatment of solutions, and commitment between diversification and intensification in the search within the space of solutions. These characteristics set genetic algorithms as the final key for solving the node localization problem.

Genetic algorithms are based on the hypothesis of the theory of evolution, where individuals better adapted to the environment survive and generate descendants, who with the passing of generations will acquire better characteristics of adaptation to the environment than their predecessors [35]. In this way, this methodology starts from an initial set of random solutions whose aptitudes will be evaluated by means of a fitness function (problem optimization function). Subsequently, genetic selection, crossing, and mutation operators will be applied to generate new possible solutions to the problem from the best ones of the previous generation. This process will be repeated until the algorithm converges or until a predetermined number of generations, resulting in the final solution to optimize the problem.

### 4.1. Coding

The coding allows for the transformation of the variables of the problem to a system in which the genetic operators can be applied. The way in which it is carried out will determine to a large extent the search capacity in the solution space and the convergence of the algorithm.

The selected coding is of a binary type, facilitating the implementation of genetic operators and diversifying the search for solutions [35]. However, the evaluation of the individuals of the population must be carried out based on the Cartesian coordinates *x*, *y*, *z* of each of the nodes, these being expressed in real numbers.

**Figure 4:**
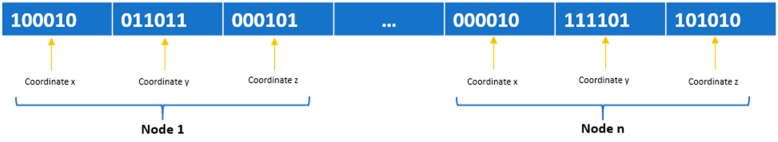
Binary coding in GA. Example of association between Cartesian node coordinates and their value in binary coding.

Figure 4 shows an example of the transformation of the Cartesian coordinates of each node into a generic individual of the population. Real coordinates based on binary are obtained in two successive steps.

First, the direct conversion between binary digits and natural numbers is carried out, based on previously defined binary chain lengths. These can be invariant for all coordinates or determined based on the maximum magnitude differences possible in the scenario and for each of the coordinates. This last option is the one finally implemented because it allows for a greater homogeneity in the spatial resolution of the solutions in the three coordinates, from 0.5 to 1 m-, and total adaptability to the NLE region limits at every point of the environment.

Secondly, each of the coordinates of the nodes is transformed into natural numbers to the values in real numbers depending on the geometry of the environment. This conversion, which is called escalation, is done through the following relationship.

(1) $$Coor_{R}=\frac{Coor_{N}}{2^{L}-1}\left(N_{max}-N_{min}\right)+N_{min}$$

where $Coor_{R}$ is the Cartesian coordinate of the node scaled in real numbers $Coor_{N}$ is the Cartesian coordinate of the node expressed in natural numbers, *L* is the length of the binary chain associated with the coordinate in question, $N_{max}$ is the maximum value of the coordinate in the scenario, and $N_{min}$ is the minimum magnitude of the coordinate in the scenario.

This real-binary conversion must be carried out by means of a sequential calculation of the Cartesian coordinates of each of the nodes present in each individual of the population. In this way, first, the *x* coordinates of each node are scaled based on the knowledge of the maximum dimensions of the scenario (invariants for the *x* coordinate). Next, the coordinates are scaled and based on the spatial limitations for the direction and the scenario based on the previously scaled *x* coordinate. Finally, the *z* coordinates are scaled according to the maximum dimensions of the environment for the *z*-direction that is dependent on the previously scaled *x* and *y* coordinates.

The use of this methodology in any environment is linked to the implementation of some type of 1D interpolation for the scaling of the coordinates, as well as to the use of 2D interpolation for the scaling of the *z*-coordinates.

The real-binary transformation is governed by the same principles, with the proviso provision that the initial real coordinate must be a multiple of the step assigned to that scaling, where the step is expressed by the following equation:

(2) $$Step=\frac{N_{max}-N_{min}}{2^{L}-1}$$

### 4.2. Selection

The choice of genetic selection operator was based on a comparison between the most widespread in the literature: Tournament 2, Tournament 3, and Roulette [36]. In addition, the comparative is completed with an analysis of elitism percentages for each selection procedure, searching for those that maximize the fitness function value while reducing the number of generations needed to reach convergence.

The results of the optimization are shown together with the number of generations needed in Scenarios 1 and 2, subject to the maximization of the fitness function and convergence criteria presented in Section 6. A number of 16 consecutive optimizations with the three operators has been performed in each scenario in order to avoid local maximums that could be achieved with this heuristic technique. This procedure allows for comparing the selection techniques in order to select the best fit for this problem.

Table 1 and Table 2 show that the best results are obtained for a selection technique of Tournament 3 in Scenarios 1 and 2, being the option chosen for the final implementation of the GA. Tournament 2 presents more stability but does not reach the same maximum values which are the final objective of the optimization.

**Figure 5:**
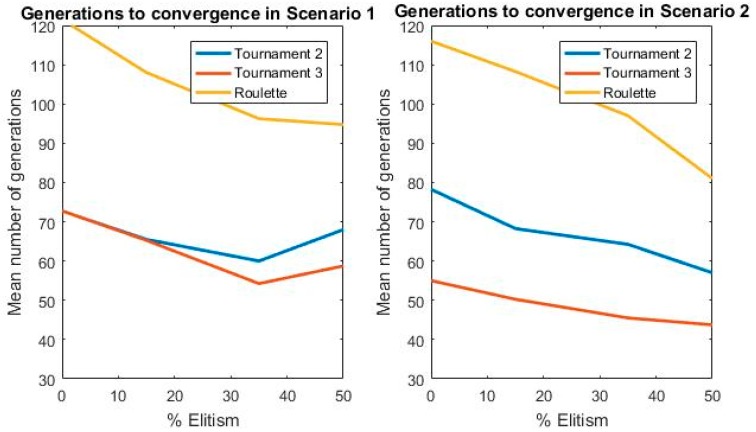
Convergence analysis in terms of elitism. In this picture, the number of generations that is needed to reach convergence is presented in Scenarios 1 and 2 in function of the percentage of elitism.

Furthermore, the number of generations to reach the convergence of the algorithm is significantly reduced with Tournament 3, as can be seen in Figure 5, because a more competitive way in the selection of the individuals is performed. Roulette has been totally discarded because the number of generations suffers a notable increase which causes a computational time addition and the values of the optimization present more instabilities than in the other techniques.

For these reasons, Tournament 3 was the final selection operator choice. Then, elitism had to be selected. Subsequently, a comparison was made for different percentages of elitism in the selected strategy, Tournament 3.

Based on Table 1, Table 2 and Table 3 it is possible to conclude that the genetic operator of selection most appropriate to this problem is Tournament 3 with 15% elitism, which is a compromise between a competitive selection and conservation of the best individuals that maximize the fitness function.

### 4.3. Crossover and Mutation

The genetic operator of the selected crossing has been a single-point crossover. In conjunction with the selection strategy implemented, it provides an appropriate behavior as a compromise between rapid convergence and the ability to prevent local optimums as final results. As for the mutation, a 4% probability has been selected to facilitate the initial diversification in the search for solutions without harming intensification in the optimal region of solutions.

## 5. Fitness Function and Algorithm Convergence

Genetic evolution of the generations is conditioned to an analysis of the beauty of each individual through a fitness function. In this problem, the CRLB estimator has been selected to perform the optimization of the sensor location in an asynchronous positioning architecture (A-TDOA) [6,7]. CRLB allows obtaining the minimum value of the global positioning error.

CRLB is an unbiased estimator of the lowest variance of a determining parameter. In positioning systems, CRLB determines the minimum error in the calculation of the position by any algorithm used under both LOS and NLOS conditions. CRLB estimates the variance of the position estimation by means of the Fisher Information Matrix (FIM):

(3) $$var\left(\hat{\theta }\right)\geq \frac{1}{FIM}=\frac{1}{E\left[{\left[\frac{\partial }{\partial x}lnln\;f\left(X;\theta \right)\right]}^{2}\right]}$$

Being $\hat{\theta }$, the unbiased estimator of the parameter under study, *E* the expectation value of the denominator function, *θ* the parameter under study and *X* the measurements of this parameter that define a probability density function f(X;θ).

A White Gaussian Noise (WGN) is modeled with an association with the uncertainties of the time measurements. The variance of the WGN depends on the distance between the emitter and the receiver of the positioning signals. This leads to the heteroscedasticity of the noises. Huang et al. [31] defined a model in order to introduce all these parameters into the inverse of the FIM (J):

(4) $$J_{mn}={\left(\frac{\partial h\left(X\right)}{\partial x_{m}}\right)}^{T}R^{-1}\left(X\right)\left(\frac{\partial h\left(X\right)}{\partial x_{n}}\right)+\frac{1}{2}tr\left(R^{-1}\left(X\right)\left(\frac{\partial R\left(X\right)}{\partial x_{m}}\right)R^{-1}\left(X\right)\left(\frac{\partial R\left(X\right)}{\partial x_{n}}\right)\right)$$

Matrix *h(X)* contains the information of the distance differences between the target and sensors, where *m* and *n* sub-indexes express the variables to estimate that are involved in the calculation of each FIM component. These differences are defined according to the A-TDOA architecture [7] where the positioning signal travels from the Target Sensor (TS), Coordinator Sensor (CS)—where time differences are processed—and the Worker Sensors (WS)—where positioning signals are emitted to the Target Sensor [6]:

(5) $$h_{i}=\|TS-WS_{i}\|+\|TS-CS\|-\|WS_{i}-CS\|\;i=1,\ldots ,\;N_{WS}$$

where *N_WS_* is the number of WS nodes. *R(X)* is the covariance matrix, where the CRLB variance definition is implemented according to a noise model characterization based on Log-normal path loss propagation model [37]:

(6) σi2=c2B2(PTPn)PL(d0)[(did0)n+(dTSd0)n+(dCSd0)n]i=1,…, N

where c is the signal propagation speed in m/s, B is the signal bandwidth in Hz, $P_{T}$ is transmission power, $P_{n}$ is the mean noise power that is modeled according to Johnson-Nyquist relation, n is the path loss exponent, $d_{0}$ is the reference distance between emitter and receiver from which Log-normal model is correctly implemented and $PL\left(d_{0}\right)$ is the path loss associated with $d_{0}$. Distances $d_{i}$, $d_{TS}$ and $d_{CS}$ are expressed by the following equations.

(7) $$d_{i}=\|TS-WS_{i}\|\;d_{TS}=\|TS-CS\|\;d_{CS}{}_{i}=\|WS_{i}-CS\|\;i=1,\ldots ,\;N$$

The Root Mean Square Error (RMSE) measures the uncertainty of the sensor location. In this model, RMSE can be obtained through the terms of the main diagonal of the inverse of the Fisher Information Matrix:

(8) $$RMSE=\sqrt{trace\left(J^{-1}\right)}$$

The analysis of the RMSE in each point of the TLE for Scenarios 1 and 2 has been defined as the fitness function in order to evaluate the quality of the sensor distributions in the A-TDOA architecture. The high amount of analysis points has led to a consideration of the mean values of the RMSE all over the TLE. The measure of the RMSE for each individual in the GA defines the best candidates to achieve the optimization, being the best individuals directly conserved for the next generation through elitism principles defined in Section 4.2.

The converge criterion of the algorithm has been established by means of the stagnation of the maximum value of the fitness function in a number of consecutive generations. Furthermore, a number of coincidences among the individuals in the population of the last generation must be assured to stop the optimization process. This configuration allows a trade-off solution between the exploration in the space of solutions and the total processing time. Both Scenarios 1 and 2 present the best optimization results when a convergence criterion of the 80% of the individuals is considered.

## 6. Results

This section presents the results of optimizations for CRLB in an A-TDOA positioning system in Scenarios 1 and 2. Initially, a series of communication parameters linked to the positioning architecture have been defined in Table 4.

The simulations presented below have been obtained based on an algorithm configuration with the Tournament 3 type selection strategy, elitism of 15%, single-point crossing, mutation probability of 4%, and binary coding with the scaling of individuals. All the optimizations have been made based on a total of 5 sensors to perform the position calculation, minimum necessary from the mathematical point of view [11] to perform 3D localization. The computational complexity of the algorithm is a polynomic order $\left(O\left(n^{a}\right)\right)$ where super-index a is highly variable due to its dependency on the size of the TLE region, the GA population, and the number of generations to convergence. Algorithm coding and representation have been implemented in the MATLAB platform.

**Figure 6:**
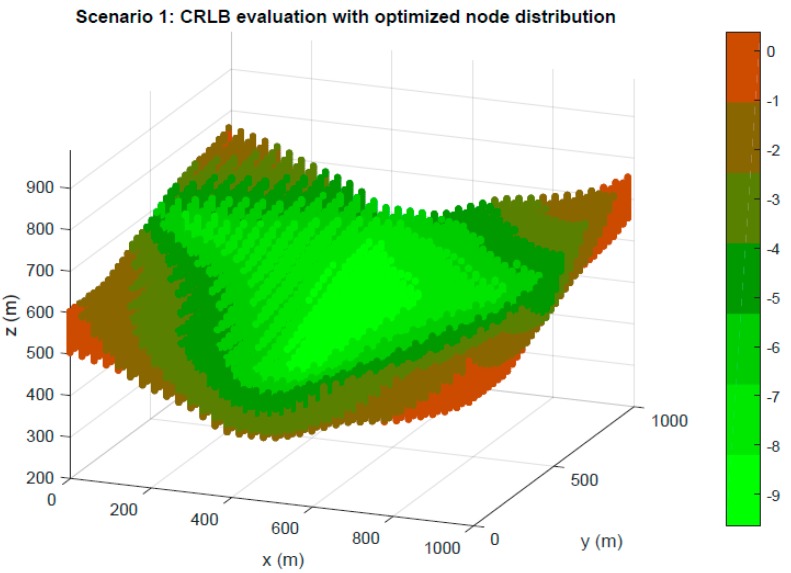
Optimization in Scenario 1. CRLB in meters for TLE region based on node location optimized by GA.

**Figure 7:**
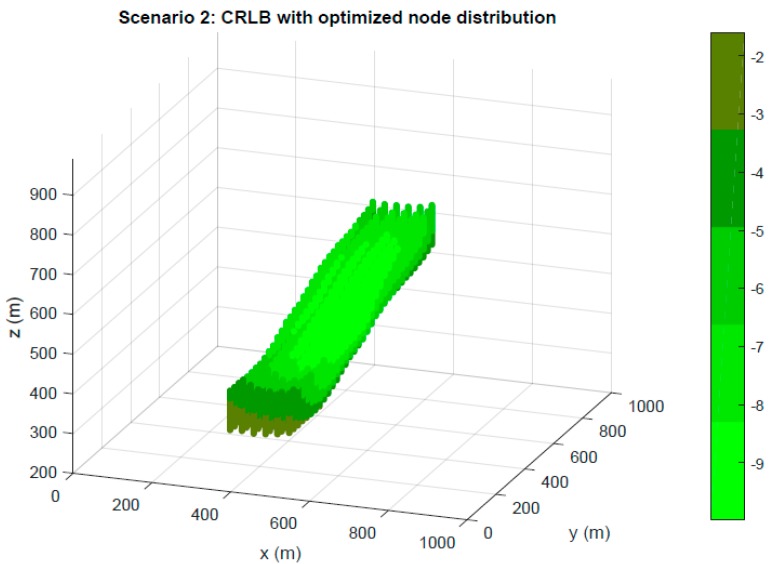
Optimization in Scenario 2. CRLB in meters for TLE region based on node location optimized by GA.

First, in Figure 6 and Figure 7, a CRLB evaluation in terms of dB in the TLE of Scenarios 1 and 2 is presented for node distributions optimized by the GA. Notable stability on the CRLB values can be observed and the results have supposed an improvement in system properties from initial random populations.

**Figure 8:**
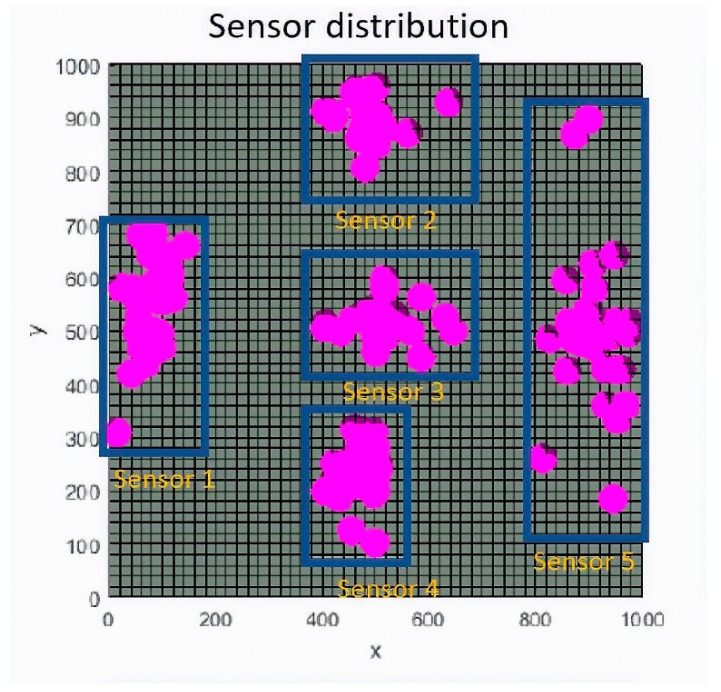
Sensor distribution in the x-y plane in meters. Each sensor defines an environment where convergence always happens during the optimization process with independence on the initial random population. In this figure the result of 48 different optimizations is represented.

In the development of a set of optimizations with random initial populations for the two scenarios, it has been observed, as in Figure 8, that whatever the initial starting population of the iterations, the convergence of each of the five beacons occurs in a very specific environment for each of them. This is due to the fact that the sensor positioning search region is very limited and shows the stability in the optimization of the genetic algorithm.

With the optimization region delimited for each one of the sensors in Figure 8, the free movement zone of the beacons (NLE) is fixed to the environment delimited by the blue rectangles for each sensor. This way, the search space is reduced, and the convergence of the algorithm is achieved beforehand. In addition, the definition of this environment allows refining the spatial grid spacing in each coordinate, in order to obtain smaller steps and thereby obtain optimizations of the location of the sensors in the space with higher resolution and better properties.

Final results of this optimization with the new solution space properties previously defined are shown and compared with a random node distribution from the initial population of the GA in Table 5 and Table 6.

These results show the possibility of using the methodology described as a sensor placement algorithm for applications with high accuracy needs in the positioning of objects. Likewise, a technique with a high degree of modularity is provided, which can be applied to any positioning architecture based on temporary measurements. Finally, the heteroscedastic characterization of the uncertainty associated with each of the temporary measurements of the system allows a high degree of correspondence with the reality, especially important in LPS systems.

## 7. Discussion

In the current article, we present the development of a genetic algorithm that allows the 3D localization of sensors in a local positioning system. This problem has special relevance since the error associated with the location of vehicles is very dependent on the spatial location of the sensors.

Specifically, the sensor layout is the sole source of controllable error once the algorithms for calculating the position, the architecture of the system (A-TDOA) and the process of measuring the magnitude that allows locating times have been established.

In particular, the spatial distribution of the sensors determines the geometrical properties of the intersection of the hyperboloids in this problem [11], thus reducing the achievable error levels. For this reason, it has been shown in the state of the art that this problem of the location of sensors has been widely studied in recent years. However, most of these articles address a two-dimensional location problem that in this article has also been extended to the third coordinate of the space to consider the flight of UAVs in local positioning systems.

In addition, these articles present very defined scenarios in indoor environments with ad-hoc resolutions for the environments designed. However, in this article, the problem-solving methodology is detailed in a generalized way for very different scenarios and contexts. This situation is particularized in two different scenarios where the modeling of the ground follows a randomness that brings the problem closer to a real context.

The studies carried out in these contexts allow us to optimize the spatial distribution of sensors for an asynchronous positioning architecture for the first time. To do this, the CRLB estimator is used to obtain the lowest error level achievable for any algorithm in the possible domain for locating the vehicles.

For this reason, this article is especially relevant for locating vehicles in precision activities in outdoor environments and for indoor navigation of automatic vehicles (AGVs). The results achieved show the benefits of the selected techniques and the importance of the problem posed.

In future work, not only the optimization to reduce the CRLB but also the minimization of the number of sensors necessary for the location in a given context will be considered. For this, it will be necessary to analyze the properties of signals and receiver noises in LOS and NLOS communications, which will lead to a multivariable optimization that will improve the properties of the LPS to be studied.

## 8. Conclusions

The high accuracy requirements in the positioning system demanded in new applications such as autonomous vehicle navigation (AGVs and UAVs) or tracking of robots in industrial plants, have led to a huge increase in LPS based on asynchronous positioning architectures, as the A-TDOA. However, the intrinsic characteristics of the LPS systems cause a great dependence between the location of the sensors of the system and the degree of accuracy achieved in the positioning.

In this article, a genetic algorithm is presented for the sensor distribution in a 3D environment with total flexibility in both the definition of the optimization spaces for the location and the regions of possible location of the components of the positioning architecture. This technique has been applied to an LPS with A-TDOA architecture, although its implementation is possible with any type of positioning system. The optimization carried out was based on the CRLB, with a WGN representation in the temporary measurements. This modeling allows heteroscedasticity in the variances associated with the temporal estimation of the sensors, which constitutes a fundamental factor in order to achieve a correct representation of reality in LPS systems.

The designed algorithm provides an optimal result for the solution of this problem since it defines a location environment for every sensor in the space of each executed simulation, showing great stability independently of the initial random population that is generated.

The definition of this solution space for each sensor allows us to reduce the search space of the solution, increasing the speed in the convergence and refining the discretization step in each coordinate in order to obtain a better resolution in the optimization.

## Figures and Tables

**Table 1:** Selection technique analysis. Mean and maximum fitness function values in Scenario 1 for Tournament 2, Tournament 3, and Roulette.

Selection Technique	Mean Fitness Function	Max Fitness Function
Tournament 2	646	656
Tournament 3	643	658
Roulette	618	649

**Table 2:** Selection technique analysis. Mean and maximum fitness function values in Scenario 2 for Tournament 2, Tournament 3, and Roulette.

Selection Technique	Mean Fitness Function	Max Fitness Function
Tournament 2	757	776
Tournament 3	753	779
Roulette	708	758

**Table 3:** Maximum fitness function representation for the best selection operator (Tournament 3) in terms of elitism percentage during population reproduction.

Elitism	Max Fitness Function Scenario 1	Max Fitness Function Scenario 2
0%	655	778
15%	658	779
35%	649	752
50%	647	749

**Table 4:** A-TDOA system communication parameters for optimization. Their election has been made based on aeronautical tracking applications [38], with the objective of representing the use of generic technology in the CRLB analysis.

Parameter	Value
Transmission power	400 W
Mean noise power	−94 dBm
Frequency of emission	1090 MHz
Bandwidth	100 MHz
Path loss exponent	2.1
Antennae gains	Unity
Time-Frequency product	1
Communication type	Full-duplex

**Table 5:** Final results. CRLB statistics in Scenario 1 for random and optimized node distributions of 5 A-TDOA sensors.

Scenario 1	Random Node Placement	Optimized Node Placement
Mean (m)	1.759	0.432
Max (m)	19.940	1.089
Min (m)	0.131	0.109
% < 0.5 m	11.44%	65.43%

**Table 6:** Final results. CRLB statistics in Scenario 2 for random and optimized node distributions of 5 A-TDOA sensors.

Scenario 2	Random Node Placement	Optimized Node Placement
Mean (m)	3.271	0.261
Max (m)	34.611	0.693
Min (m)	0.316	0.101
% < 0.5 m	6.54%	94.56%
